# YAP manipulates proliferation via PTEN/AKT/mTOR-mediated autophagy in lung adenocarcinomas

**DOI:** 10.1186/s12935-020-01688-9

**Published:** 2021-01-07

**Authors:** Wei Xu, Mingjiong Zhang, Yue Li, Yu Wang, Kai Wang, Qiaoyu Chen, Runjie Zhang, Weiwei Song, Qiqing Huang, Weihong Zhao, Jianqing Wu

**Affiliations:** 1 Jiangsu Provincial Key Laboratory of Geriatrics, Department of Geriatrics, The First Affiliated Hospital of Nanjing Medical University, Nanjing, China; 2 Center for Reproductive Medicine, Shanghai First Maternity and Infant Hospital, Tongji University School of Medicine, Shanghai, China; 3 Zhongda Hospital Lishui Branch, Nanjing Lishui People’s Hospital, Southeast University, Nanjing, China

**Keywords:** LUADs, YAP, PTEN/AKT/mTOR, Autophagy

## Abstract

**Background:**

Autophagy is a double-edged sword during the initiation and progression of multiple tumors. The Hippo pathway effector YAP has been proved to be involved in autophagy processes. The present study aimed to investigate how YAP regulates cell proliferation via autophagy in lung adenocarcinomas (LUAD).

**Methods:**

Data of LUAD chip GSE43458 was obtained from Gene Expression Omnibus (GEO). RT-qPCR and Western blot were performed to assess YAP expression in LUAD cell lines. CCK-8 assay, xenograft tumor model, immunochemistry and GFP-mRFP-LC3 fusion proteins were utilized to evaluate the effect of YAP on autophagy of LUAD cells *in vitro* and *in vivo*. Autophagy inhibitor treatment and rescue experiments were carried out to elucidate the mechanism by which YAP manipulates autophagy in LUAD cells.

**Results:**

YAP was significantly overexpressed in samples of LUAD patients and its expression level is related to 5-year survival. YAP manipulated the proliferation and autophagy in A549 and H1299 LUAD cells. YAP could induce activation of Akt/mTOR signaling pathway via suppressing PTEN in a Hippo-pathway-dependent manner. 3-Methyladenine impeded autophagy flux and promoted the proliferation *in vitro* and *in vivo*.

**Conclusions:**

Hippo pathway critical transcriptional coactivators YAP manipulates the proliferation of lung adenocarcinoma, which is regulated by PTEN/AKT/mTOR autophagic signaling.

## Introduction

Yes-associated protein (YAP, also known as YAP1) is a downstream transcriptional coactivator of the Hippo pathway, which plays a key role in embryogenesis, tissue homeostasis and organ regeneration [1, 2]. The Mammalian STE20-Like Protein Kinase 1/2 (MST1/2) and Large Tumor Suppressor Kinase 1/2 (LATS1/2) are core serine/threonine kinases of the Hippo pathway, expedited by scaffold proteins such as Salvador Family WW Domain Containing Protein 1 (SAV1) and Mps One Binder Kinase Activator-Like 1A/B (MOB1A/B). Upon activation of the Hippo pathway, LATS1/2 kinases are phosphorylated, which in turn phosphorylate YAP. Phosphorylation of YAP triggers proteasomal degradation by binding to 14-3-3 proteins or b-TrCP in the cytoplasm [3, 4]. When the Hippo pathway is inactivated, YAP is translocated into the nucleus. As a transcriptional coactivator, YAP binds to TEA domain family member (TEAD) to stimulate a gene transcription profile involved in cell proliferation, differentiation and metabolism [5].

In addition to its physiological functions during tissue repair and organ regeneration, there are accumulating evidences shown that overexpression of YAP plays an important role in oncogenesis of a variety of cancer types [6–8]. Though hardly detectable in normal cells, aberrantly overexpression of YAP have been reported in several malignant cancers including breast cancer, lung cancer, glioblastoma, and pancreatic cancer [6, 9]. YAP stimulates tumor cell proliferation by amplifying the expressions of oncogenes, such as MYC and AP-1 family members (JUN and FOS-like factors), thus affecting DNA duplication, DNA repair and mitosis in cancers [7, 10, 11]. Overexpression of YAP induces the immortalized cells to gain stemness properties along with the appearance with epithelial-to-mesenchymal transition (EMT) phenomenon [12, 13]. A great number of researches have indicated that YAP is an indispensable element required by the steps of the invasion-metastasis cascade [14]. The above pathological process has been demonstrated to be based on multiple signaling pathways such as RAS–PI3K pathway, or the WNT–APC–AXIN pathway, in which YAP functions as a key effector. Lung adenocarcinoma (LUAD), accounting for approximately 40% of lung cancers with poor prognosis, is reported to highly express YAP, which is relative to the rapid progression according to the latest studies [15, 16]. However, the mechanism of YAP in initiation and progression of LUAD is still unclear, which hinders the exploitation of antineoplastic strategies.

Macroautophagy (referred to throughout this article as autophagy) is a highly conserved catabolic process that devours cellular abnormal proteins and damaged organelles into the lysosome to maintain cellular environmental homeostasis [17–19]. The dysregulation of autophagy has been shown to regulate several intracellular events in cancers [20–22]. Autophagy is a double-edged sword as it can both prevent the initiation and on the other hand accelerate the progression of multiple tumors [23] .And its complex function can be related to multiple biological factors such as different tumor types and cancer genotypes. Mandelbaum et al. have identified that the lung cancer cell line H1650 is deficient in ATG7-dependent autophagy. Later, Gurpinar et al. found a novel sulindac derivative which inhibits lung adenocarcinoma cell growth through induction of autophagy [20, 24]. However, Pan et al. has shown that HMGB1-mediated autophagy promotes docetaxel resistance in human lung adenocarcinoma and is required for progression by malignant tumors [25].

Recently, YAP has been proved to be involved in autophagy processes in many diseases, such as the hepatic carcinogenesis, osteogenesis, tuberous sclerosis complex delete [26–28]. Therefore, identifying the important role of YAP in malignant tumors may provide new therapeutic approaches for tumors with high YAP expression. In addition, the relationship between YAP and autophagy in the occurrence and development of LUAD remains unclear. Here, we explored the function of YAP in the process of autophagy in LUAD. Our findings would provide a potential idea for Yap-targeted therapy in LUAD.

## Materials and methods

### Data resource

Data of LUAD chip GSE43458 was obtained from Gene Expression Omnibus (GEO) (http://www.ncbi.nlm.nih.gov/geo/). GEO query package was used to download the data of YAP expression. Fifty LUAD tissues and twenty-five normal lung tissues were obtained from patients at dataset GSE43458. Gene expression profiling was performed on the samples to identify differentially expressed profiles between lung adenocarcinomas and normal lung tissues. Meanwhile, gene expression profiles and protein expression profiles of LUAD were obtained from TCGA database (https://portal.gdc.cancer.gov) and Human Protein Atlas database (https://www.proteinatlas.org), including clinicopathological features like patient’s age at diagnosis, gender, race, diagnostic classification, tumor stage, tumor grade and 5-year survival.

### Cell culture and reagents

Human LUAD cells A549 and H1299 were purchased from the Chinese Academy of Sciences (Shanghai, China). A549 and H1299 cells were cultured in RPMI1640 medium (Gibco, USA) containing 10% fetal bovine serum (Invitrogen, USA), 100 U/ml penicillin and 100 µg/ml streptomycin in a humidified atmosphere of 5% CO_2_ at 37 ℃. All the cell lines in this study were regularly confirmed for the absence of Mycoplasma contamination. 3-Methyladenine (3-MA) was purchased from Selleck (Houston, TX, USA) and used as the figure legend indicated.

### DNA plasmids, siRNA, and shRNA sequences and transfection

Human gene expression plasmids pcDNA3.1-YAP, pcDNA3.1-PTEN, gene-specific siRNAs, gene-specific shRNAs and control were from GenePharma (Shanghai, China). The target sequences were as follows: shPTEN1: GTCTGACCTAGTTAATTTACA; shPTEN2: GCAGGCTTCCAAAGGCTTATG; siYAP1: CUGCCACCAAGCUAGAUAATT; siYAP2: GCCAGUACUGAUGCAGGUATT; siLATS: GGUAGUUCGUCUAUAUUAUTT. Transfection of plasmids into A549 and H1299 cells were carried out using Lipofectamine 2000 (Invitrogen, USA) according to the manufacturers’ instructions. Lipofectamine RNAiMAX (Invitrogen, USA) was used to transfect siRNAs.

### Cell proliferation assay

Cell Counting Kit-8 (CCK-8, Beyotime, Jiangsu, China) was applied to measure cell proliferation. Transfected cells were plated in 96-well plates (1500 cells/well) and cultivated for 4 days. In the drug treatment group, after 10 hours post incubation to allow cell adherence, 3-MA was applied at the concentration of 2 mM. In each well, 10 µl of CCK-8 reagent was added, followed by 1.5 h of incubation at 37 °C. The results were measured by the absorbance at 450 nm. All experimental points were performed with 6 to 8 duplicate wells, and all experiments were performed at least 3 times.

### Western blotting

Total protein was harvested after cell treatment, and the protein concentration was determined by the BCA protein assay kit (Beyotime, Jiangsu, China). 15 µg of lysate was separated via 10–15% SDS-PAGE (Beyotime, Jiangsu, China), proteins were then transferred onto a PVDF membrane. After blocked with 5% defatted milk, the membranes were incubated with primary antibodies at 4 °C overnight and with HRP-conjugated IgG at room temperature for 2 h. Antibodies specific for YAP (1:1000, #14,074,Cell Signaling Technology, Boston, MA, USA), LC3 I/II (1:1000, #3868T, CST, USA), p62 (1:1000, #5114T, CST, USA), PTEN (1:1000, #9559T, CST, USA), phospho-AKT (1:1000, #4060T, CST, USA), AKT (1:1000, #4691T, CST, USA), phospho-p70S6K (1:1000, ab131436, abcam, USA), p70S6K (1:1000, #2708T, CST, USA), and Lats (1:1000, #3477s, CST, USA) were applied according to the manufacturer’s recommendations. An antibody specific for β-actin (1:500, BM0627, Boster, USA) was used as an internal control. Chemiluminescence was detected with X-Ray films or Fusion FX (Analis).

### Immunofluorescence analysis

We planted cells onto glass coverslips and fixed in a 4% paraformaldehyde solution for 20 min at room temperature. After PBS wash, the cells were permeabilized with 0.5% Triton X-100 for 20 min. Following another washing with PBS, cells were blocked for 30 min at room temperature with 5% BSA solution. The phospho-AKT, phospho-p70S6K and YAP antibodies were diluted in 1% BSA and incubated on cells at 4 °C overnight. The coverslips were washed 3 × with PBS and placed in Dylight488 (1:200, A232300, Abbkine) for 30 min at 37 °C, in the dark. The cells were washed 3 × with PBST and counterstained with DAPI for 5–10 min at room temperature in the dark. All images were obtained using 200 × magnification on a Zeiss Axioplan microscope equipped with a Zeiss camera and software.

### mRFP-GFP-LC3 detection

For autophagic flux measurements, cells cultured on 34-mm glass coverslips were transfected with the mRFP-GFP-LC3 tandem construct. After 24 h of expression, the coverslips were observed with a laser scanning confocal microscope (OLYMPUS FV-1000). The confocal images were captured at 400 × magnification to demonstrate the formation of GFP-LC3 and/or RFP-LC3 dots, which indicated autophagosomes and/or autolysosomes. The number of the fluorescent dots was counted by manual from at least three independent experiments. At least 20 cells were scored in each experiment.

### Tumor xenograft model

Five-week-old male BALB/c nude mice were purchased from Nanjing University-Nanjing Institute of Biomedicine. The animal license number was SCXK (Jiangsu) 2018-0008. The care and use of all experimental animals complied with the Nanjing Medical University (NJMU) Institutional Animal Care and Use Committee. All nude mice were housed in a specific pathogen free (SPF) grade laboratory with a constant temperature (22–25 °C) and humidity (55 ± 5%). A549 cells were transfected with siRNAs targeting YAP (siYAP) and scramble control (siNC) and cultured for proliferation. The mice were divided into 3 groups randomly: siNC, siYAP, and siYAP + 3-MA group. The cells at logarithmic phase were resuspended in serum-free 1640 and injected subcutaneously into the flanks of each nude mice. When the tumors reached 100 mm^3^, the mice of siYAP +3-MA group were administered with 3-MA by intraperitoneal injection (25 mg/kg, once every two day, 21 days in total). Tumor size was measured with vernier calipers for the long diameter (A) and short diameter (B), and the volume was calculated with the formula: V = A × B^2^ × 0.5. The xenograft tumors were extracted, weighted and processed for western blotting and histopathologic examination. Histological serial sections (4 µm thick) of selected paraffin-embedded specimens were prepared for immunohistochemistry (IHC). The slides were incubated with primary antibodies specific for LC3I/II (1:1000, #3868T, CST, USA) and p62 (1:2000, ab207305, abcam, USA) at 4 °C overnight. Then the slides were incubated with MaxVisionTM 2 followed by the DAB chromogen and hematoxylin counterstain. Images of three randomly chosen fields from each slide were collected under a microscope for statistical analysis.

### Statistical analysis

The data were expressed as the mean ± standard deviation (SD). Student’s t test (for two groups comparison) or one-way analysis of variance (ANOVA; for more than two groups comparison) was utilized to analyze significant difference between experimental groups using Prism software (GraphPad Software, La Jolla, CA, USA). Kaplan–Meier method and log-rank test was used to assess overall survival. *P* < 0.05 was considered to be statistically significant. *P < 0.05; **P < 0.01; ***P < 0.001.

## Results

### YAP is upregulated in LUAD tissues and is associated with poor prognosis of 5-year survival

**Fig. 1:**
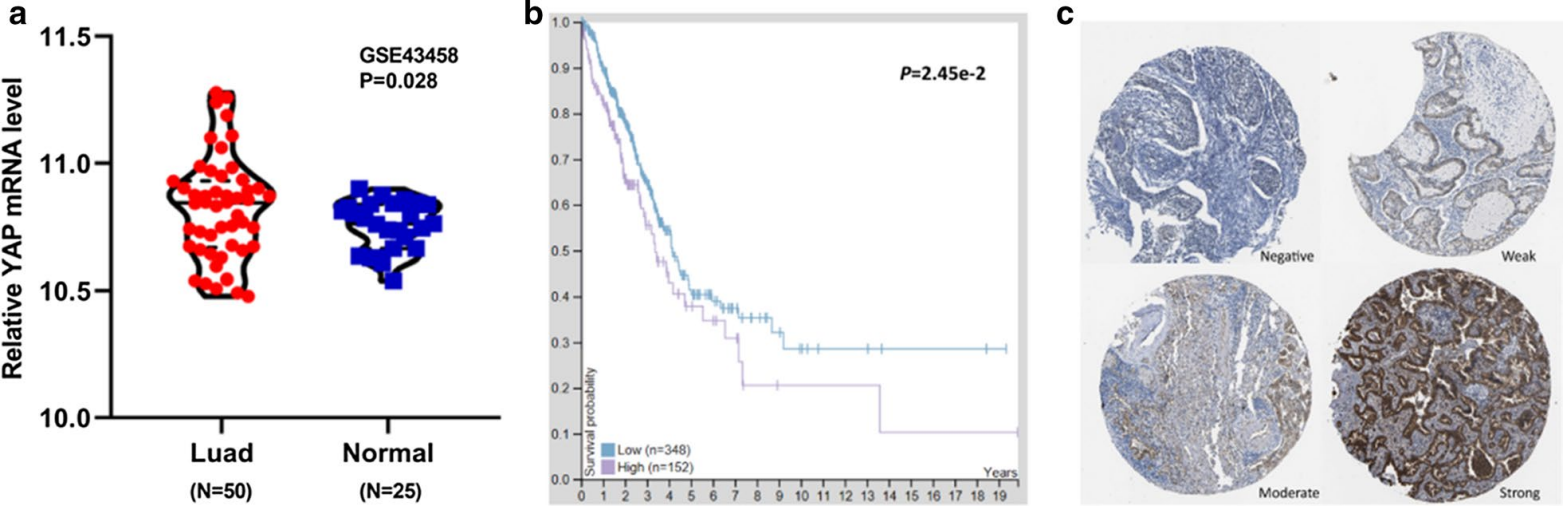
YAP is highly expressed in LUAD tissues and predicts poor prognosis. **a**. The expression of YAP in 50 tissues of LUAD and 25 tissues of normal lung tissues was determined of the GSE43458 at GEO database. **b**. Kaplan–Meier survival curves and log-rank tests were used to assess the relationship between YAP levels and overall survival time of LUAD patients. The median of YAP expression levels in LUAD tissues was taken as cutoff. **c**. YAP levels of in four LUAD tissues The Human Protein Atlas: Intensity (negative, weak, moderate, strong) https://www.proteinatlas.org/ENSG00000137693-YAP1/pathology/lung+cancer#imid_19107997

We investigated YAP expression data gathered from tissue specimens of patients with lung adenocarcinomas and normal lung tissues from GEO database. Independent samples t-test performed for GSE43458 data, the result showed that YAP mRNA expression of LUAD tissues was significantly higher than that of normal lung tissues (*P* = 0.028, Fig. 1a). Additionally, 348 cases of LUAD tissues and 152 cases of normal tissues were obtained from the TCGA database to confirm the YAP expression by the microarray data.

As shown in Table 1, YAP expression was significantly associated with gender (*P* = 0.006) and tumor stage (*P* = 0.046), which was no significant correlation with age (*P* = 0.525) in LUAD. Log-rank *P*-value for Kaplan-Meier plot showed that YAP mRNA expression level was correlated with 5-year survival (*P* = 0.025, Fig. 1b). The Human Protein Atlas also contained images of histological sections from normal lung and LUAD tissues by immunohistochemistry. We selected representative images to show high, medium, low and not detected YAP protein expression in lung tissue samples.

**Table 1:** The character of YAP expression among the different groups in lung adenocarcinoma patients at The Cancer Genome Atlas (TCGA) database

Description	Number	Mean (95% CI)	P-value
Age			0.525
≤ 65	219	21.388 (-2.06 1.05)	
> 65	281	21.891 (-2.06 1.05)	
Gender			0.006
Female	270	22.620 (0.65 3.75)	
Male	230	20.425 (0.65 3.75)	
Stage			0.046
Early (I & II & IIIa)	411	21.304 (0.09 9.06)	
Advanced (IIIb & IV)	35	25.878 (0.09 9.06)	

(Fig. 1c).

## YAP manipulates the proliferation of lung adenocarcinoma cells

**Fig. 2:**
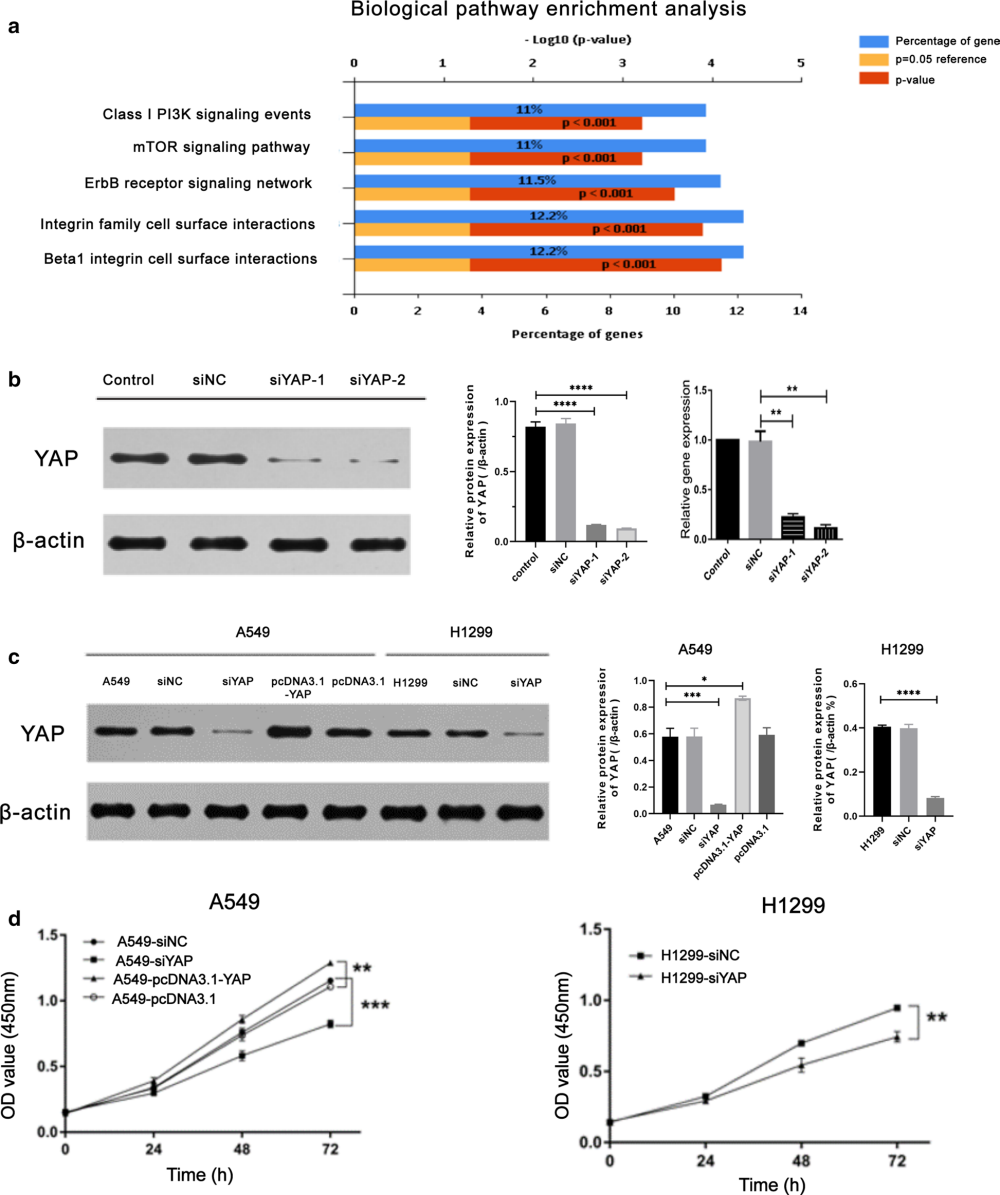
YAP targeted siRNA suppresses YAP expression in A549 and H1299 cells with reducing cell proliferation. **a** The result of biological pathway enrichment analysis was shown with respect to YAP. **b** siYAP-1/2 was constructed and significantly inhibited the expression of YAP in A549 cells, which detected by qPCR and Western blot. **c** The protein expression of YAP in A549 transfected with siYAP or pcDNA3.1-YAP, and H1299 with siYAP transfection were analysed by Western blot, respectively. **d** CCK-8 assay was utilized to analyze cell proliferation of A549 cells with siYAP or pcDNA3.1-YAP, and H1299 with siYAP transfection. Data represent the Mean ± SD on three independent experiments (one-way ANOVA, **P < 0.01, ***P < 0.001)

In order to uncover the role of YAP in lung adenocarcinoma, we firstly searched GEO database and performed biological pathway enrichment analysis utilizing GSE64550 datasets, which was designed as RNAseq analysis of HCC364 (lung adenocarcinoma) cells in the context of shRNA knockdown of the gene *YAP*. The biological pathway enrichment analysis identifies PI3K, mTOR and ErbB related biological pathways (Fig. 2a). Because both PI3K and mTOR signaling pathways are known to associate with cell proliferation and autophagy, and previous studies have shown the critical role of YAP in EGFR signaling, we planned to further investigate [1].

We constructed two of small interfering RNA (siRNA) capable of knocking down YAP in lung adenocarcinoma cells A549 and H1299 using empty vector as negative controls, respectively. We chose the second siRNA for rest of the experiments due to the highest efficiency to deplete YAP in cells YAP, and named it as siYAP below (Fig. 2b). The YAP overexpression vector, pcDNA3.1-YAP, were constructed and the transfection efficiency of siYAP and pcDNA3.1-YAP was validated by Western Blotting analysis (Fig. 2b, c). We utilized CCK-8 assay to detect the effects of YAP on cell proliferation in A549 and H1299 cells. According to the results, we found that knockdown of YAP expression could inhibit the proliferation of A549 and H1299 cells. While the overexpression of YAP could promote the proliferation of A549 cells (Fig. 2d). These findings suggested that overexpression of YAP may be correlated with LUAD cell proliferation.

### Knockdown of YAP promotes the progression of autophagy

**Fig. 3:**
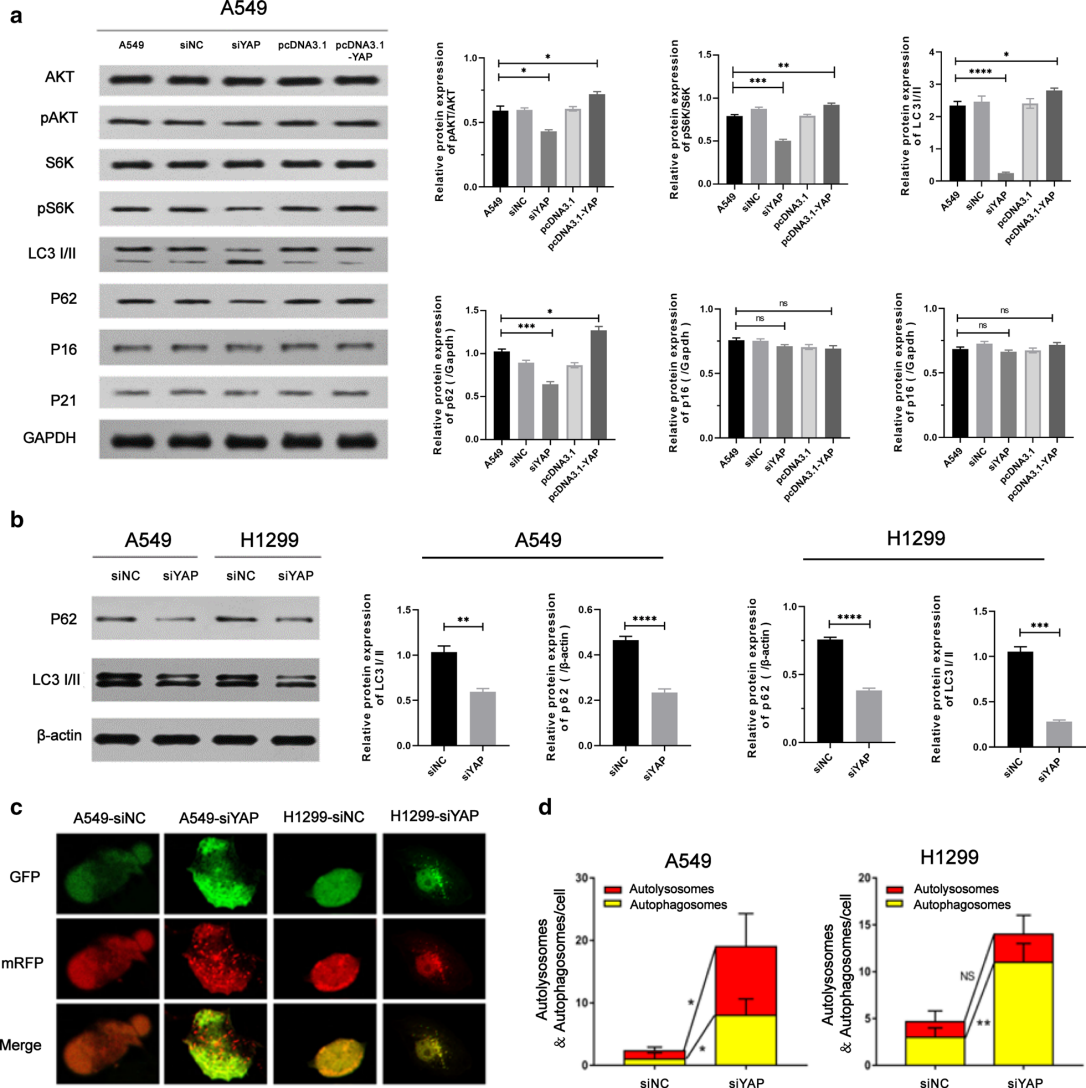
Autophagy in A549 and H1299 cell manipulated by YAP gene. **a** Analysis of autophagy-related protein expression in A549 cell manipulated by YAP1 gene. Cells were analyzed by immunoblotting with indicated antibodies. **b** Autophagy substrate proteins p62 and autophagy marker proteins LC3I/II were detected in A549/H1299-siYAP cellular extracts by Western blot. **c** Immunofluorescence analysis of autolysosomes puncta in A549 and H1299 cells transfected with siYAP. Confocal microscopy images showing cellular localization of autophagic dots in A549/H1299-siYAP cells. The GFP stain (green puncta) was on behalf of the initial process of autophagy and the mRFP (red puncta) indicated the late process of autophagy. Magnification is × 400. **d** Quantification of autophagy activity was analyzed from 10 random visual fields for each group. At least three different visual fields containing at least 20 cells were counted for each condition and shown in the graph. Data were shown as the Mean ± SD. Statistical analysis was calculated by one-way ANOVA. Scale bars = 25 µm (*P < 0.05, **P < 0.01)

According to the results of biological pathway enrichment analysis, we hypothesized that YAP regulated cell proliferation throgh autophagy process. As shown in Fig. 3a, the ratio of the autophagy marker protein LC3-II/LC3-I was significantly increased after YAP depletion. And the decrease level of phosphorylated AKT, phosphorylated S6K and p62 could also be detected. However, there were no differences in protein expression level of p16, p21, total AKT and total S6K between the different groups. On the contrary, YAP overexpression in A549 cells showed a significant reversible effect in the above changes. As LC3-I/II and p62 were important makers of autophagy, the results showed that YAP was related to the autophagy of lung adenocarcinoma cells.

Next, we focused on the relationship between YAP and autophagy in lung adenocarcinoma cells. As shown in Fig. 3b, the conversion of LC3B-I to LC3B-II in the A549 and H1299 siYAP groups was more pronounced after we knocked down YAP in both A549 and H1299 cells. Autophagic flux is also promoted by p62-mediated degradation in the cell. We found that inhibition of the YAP in A549 and H1299 cells decreased the expression of p62 (Fig. 3b). We also detected the accumulation of LC3 as autophagy process with the confocal microscopy. Confocal microscopy showed that the siYAP groups displayed a marked increase in LC3 puncta formation with a large number of red dots and green dots visible, compared with the control group where green fluorescence and red fluorescence distributed evenly in the cytosol. During excessive autophagy in A549/H1299-siYAP cells, there are more autophagosomes (yellow puncta, 8.00 ± 3.35 puncta/cell of A549-siYAP cells; 11.00 ± 3.14 puncta/cell of H1299-siYAP cells; *P* < 0.05) and more autolysosome (red puncta, 10.00 ± 4.82 puncta/cell of A549-siYAP cells; 3.00 ± 1.91 puncta/cell of H1299-siYAP cells; *P* < 0.05), compared with A549/H1299-siNC cells (Fig. 3d). The above results proved that inhibition of YAP could induce autophagy in lung adenocarcinoma cells.

#### YAP induces activation of Akt/mTOR signaling pathway via suppressing PTEN in a Hippo-pathway-dependent manner

Based on our previous study [29], we learned that S6K and AKT were direct substrates of mTORC1 and mTORC2, and YAP regulated mTOR activity. The tumor suppressor PTEN, an upstream negative regulator of mTOR, has been proved involved in the autophagy process in the occurrence and development of the malignant tumor.

**Fig. 4:**
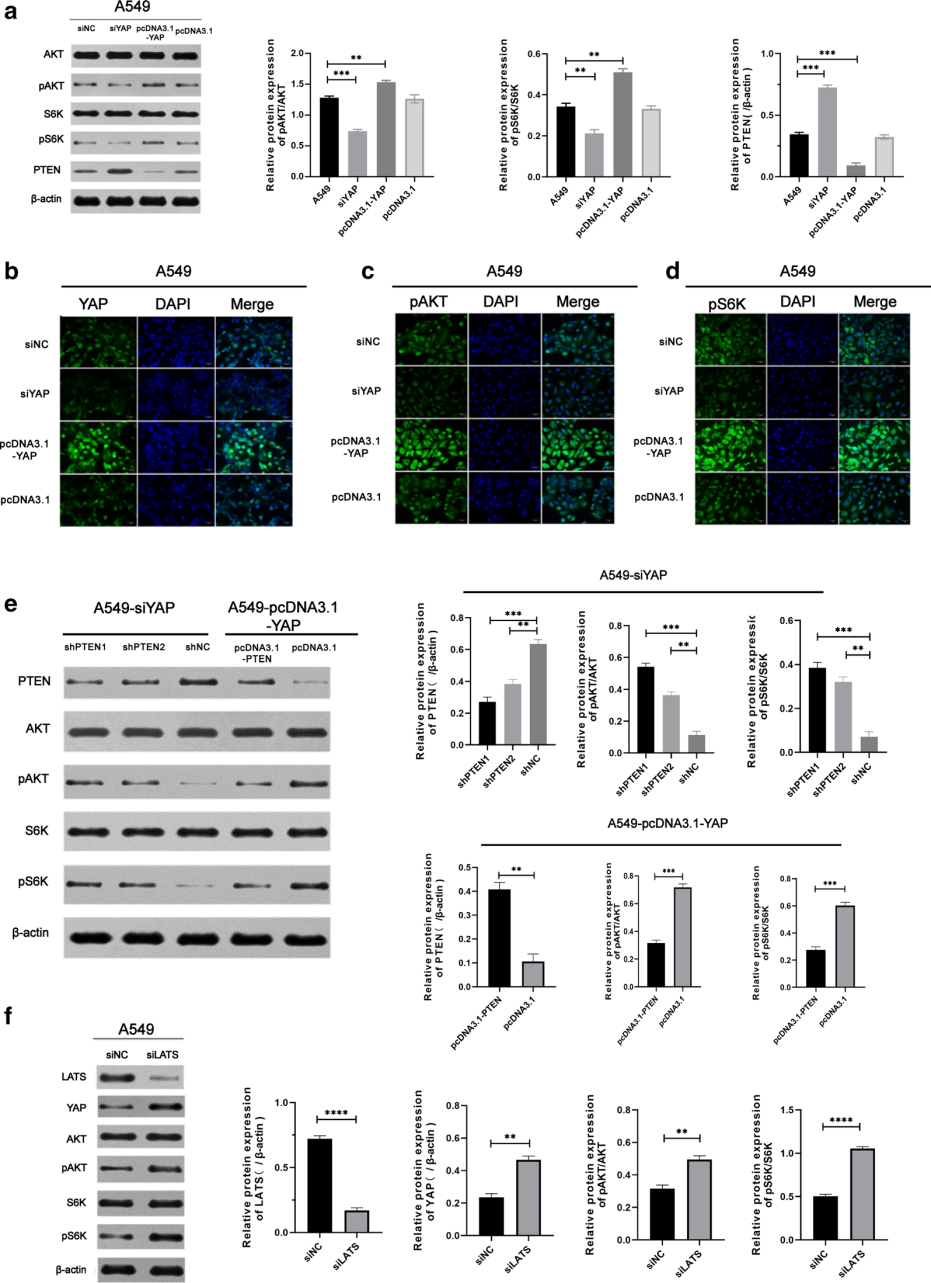
YAP induced activation of Akt/mTOR signaling pathway via suppressing PTEN in a Hippo-pathway-dependent manner. **a**. Following transfected with siYAP or pcDNA3.1-YAP in A549, Western blot measured the level of proteins associated with Akt/mTOR signaling pathway. **b**–**d** Representative immunofluorescence of A549 cells transfected with siNC, siYAP, pcDNA3.1 or pcDNA3.1-YAP was observed by Inversed Fluorescent Microscope. Fluorescent staining of YAP, pAKT and pS6K was shown as green stain and fluorescence intensity indicated the relative amount in the cells. Magnification is × 200. **e** Western blot showed the expressive levels of AKT, pAKT, S6K, pS6K, and PTEN after transfection with or without shPTEN in A549-siYAP, with or without pcDNA3.1-PTEN in A549-pcDNA3.1-YAP, respectively. **f** Western blot showed the expressive levels of AKT, pAKT, S6K, pS6K, and YAP after knockdown LATS, which being a upstream of YAP and a core factor in Hippo pathway

To investigate the molecular mechanisms of inducing autophagy after downregulation of YAP, we analyzed autophagy markers in A549 cells. The results showed overexpression of YAP increased phosphorylation of both S6K and AKT. Conversely, phosphorylation of S6K and AKT was decreased in A549-siYAP cells, where the expression of PTEN displayed an opposite trend (Fig. 4a). Immunofluorescent staining of YAP, pAKT and pS6K in A549 cells were observed by fluorescence microscope. The transfection efficiency of the siRNAs and plasmids were validated in Fig. 4b. In A549-siYAP cells, we observed that fluorescence staining of pAKT and pS6K was significantly lower than that in A549-pcDNA3.1-YAP cell (Fig. 4c, d). It is well-known that the degradation of phospho-mTOR could induce autophagy [30]. All the above results indicated that YAP reduction could induce autophagy by inhibiting activation of the Akt/mTOR signaling pathway.

To further investigate whether YAP could affect mTOR pathway by PTEN directly, we constructed the short hairpin RNA (shRNA) and pcDNA3.1 of characteristic PTEN sequence to transfected into A549-siYAP and A549-pcDNA3.1-YAP cell. In A549-siYAP cells co-transfected with shNC, the level of PTEN was higher than shPTEN group. And in A549-pcDNA3.1-YAP cells co-transfected with pcDNA3.1, the level of PTEN was lower than pcDNA3.1-PTEN group. These were in accordance with the results described in Fig. 4a. In addition, the phosphorylation levels of AKT and S6K were significantly increased in shPTEN-treated A549-siYAP cells, while decreased in pcDNA3.1-PTEN-treated A549-pcDNA3.1-YAP cells compared with the control, (Fig. 4e). Taken together, these findings demonstrated that the activity of mTOR was influenced by the expression of YAP via PTEN in lung adenocarcinoma.

YAP is considered as the core factor in Hippo pathway [8]. To confirm if autophagy was mediated by Hippo pathway, we knocked down the upstream YAP kinase LATS to detect the changes. As is shown in Fig. 4f, the expression of YAP was increased when LATS was knocked down. And the level of phosphor-AKT and phosphor-S6K were raised as well. These results indicated that autophagy was induced by Hippo-pathway-dependent pathway.

#### **3-Methyladenine impeded autophagy flux in A549/H1299-siYAP cells and promoted the proliferation**

**Fig. 5:**
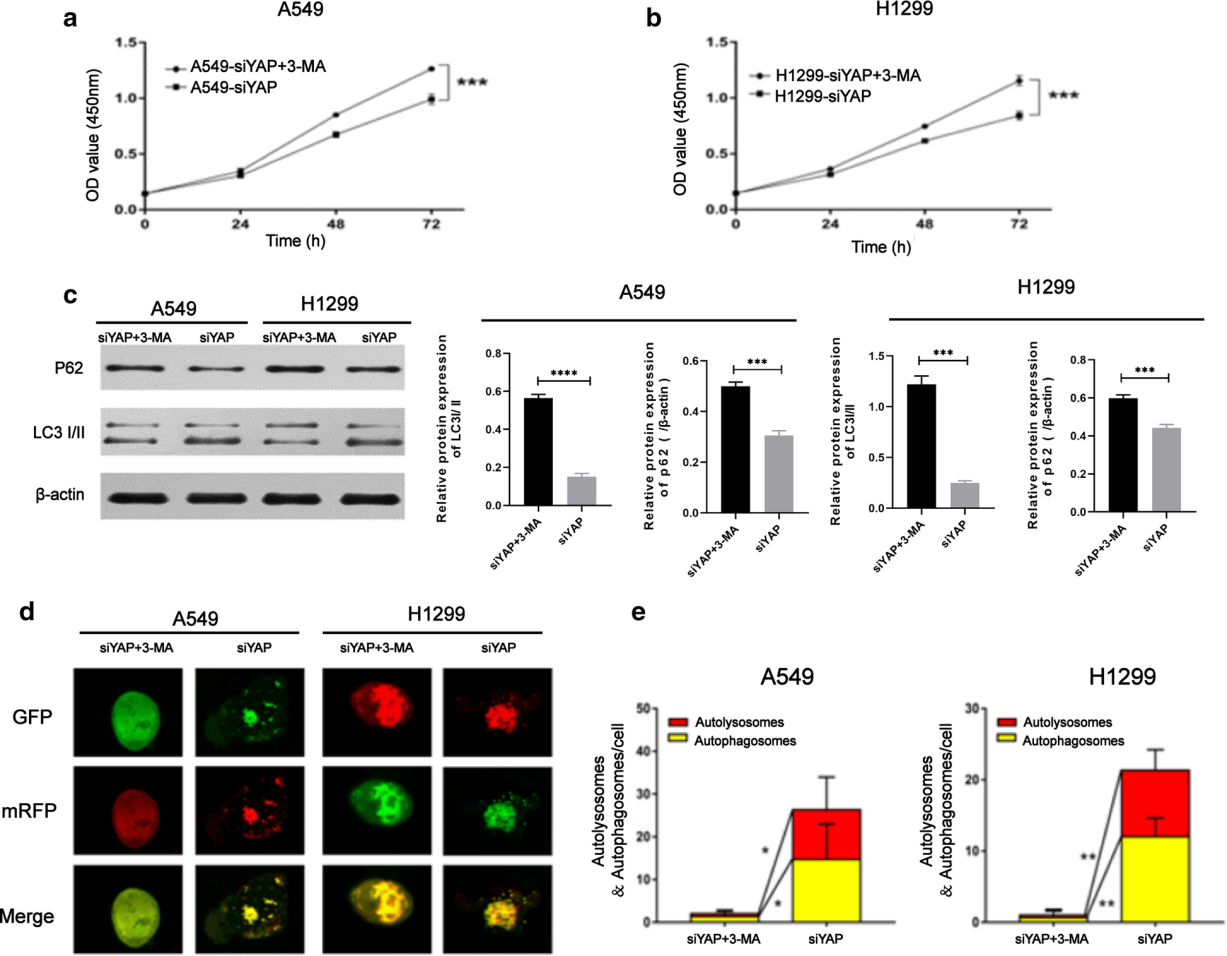
The transformation of autophagy in A549/H1299-siYAP after treated with 3-MA. **a**, **b** Theproliferationability of A549/H1299-siYAP and A549/H1299-siYAP+3-MA was determined by CCK-8 assay. **c** A549/H1299-siYAP cells treated with 3-MA were lysated. Autophagy substrate proteins p62 and the conversion of LC3B-I to LC3B-II were detected by the Western blot. **d**, **e** Confocal microscopy images showing cellular localization of autophagic dots in A549/H1299-siYAP or A549/H1299-siYAP + 3MA cells. The GFP stain (green puncta) was on behalf of the initial process of autophagy and the mRFP (red puncta) indicated the late process of autophagy. Magnification is × 400. Quantification of autophagy activity was analyzed from 10 random visual fields for each group and shown in the graph. Data were shown as the Mean ± SD (one-way analysis, *P < 0.05, **P < 0.01, ***P < 0.001)

It has been reported that autophagic-lysosomal degradation can be inhibited by 3-Methyladenine (3-MA) to impeded autophagy flux. Previous results demonstrated that knockdown of YAP could inhibit the proliferation of lung adenocarcinoma cells. At this stage, we applied 3-MA to inhibit autophagy in A549/H1299-siYAP cells, and the cell proliferation rate was significantly higher than that of the control group (*P* < 0.001, Fig. 5a, b). The expression of p62 was increased in A549/H1299-siYAP cells after 3-MA treatment, and the conversion of LC3B-I to LC3B-II was decreased compared with A549/H1299-siNC cells (Fig. 5c).

More autophagosomes (yellow puncta, 15.00 ± 5.21 puncta/cell of A549-siYAP cells; 13.00 ± 5.74 puncta/cell of H1299-siYAP cells; *P* < 0.05) and autolysosome (red puncta, 10.00 ± 5.36 puncta/cell of A549-siYAP cells; 8.00 ± 3.08 puncta/cell of H1299-siYAP cells; *P* < 0.05) were detected with laser confocal microscopy in A549/H1299-siYAP cells, compared with A549/H1299-siYAP cells treated with 3-MA (Fig. 5d). These results suggested that the downregulation of YAP induced autophagy and the possibility that YAP-dependent tumor proliferation may be partly related to the inhibition of autophagy.

#### **3-Methyladenine (3-MA) induces A549-siYAP cell proliferation and inhibits cell autophagy*in vivo***

**Fig. 6:**
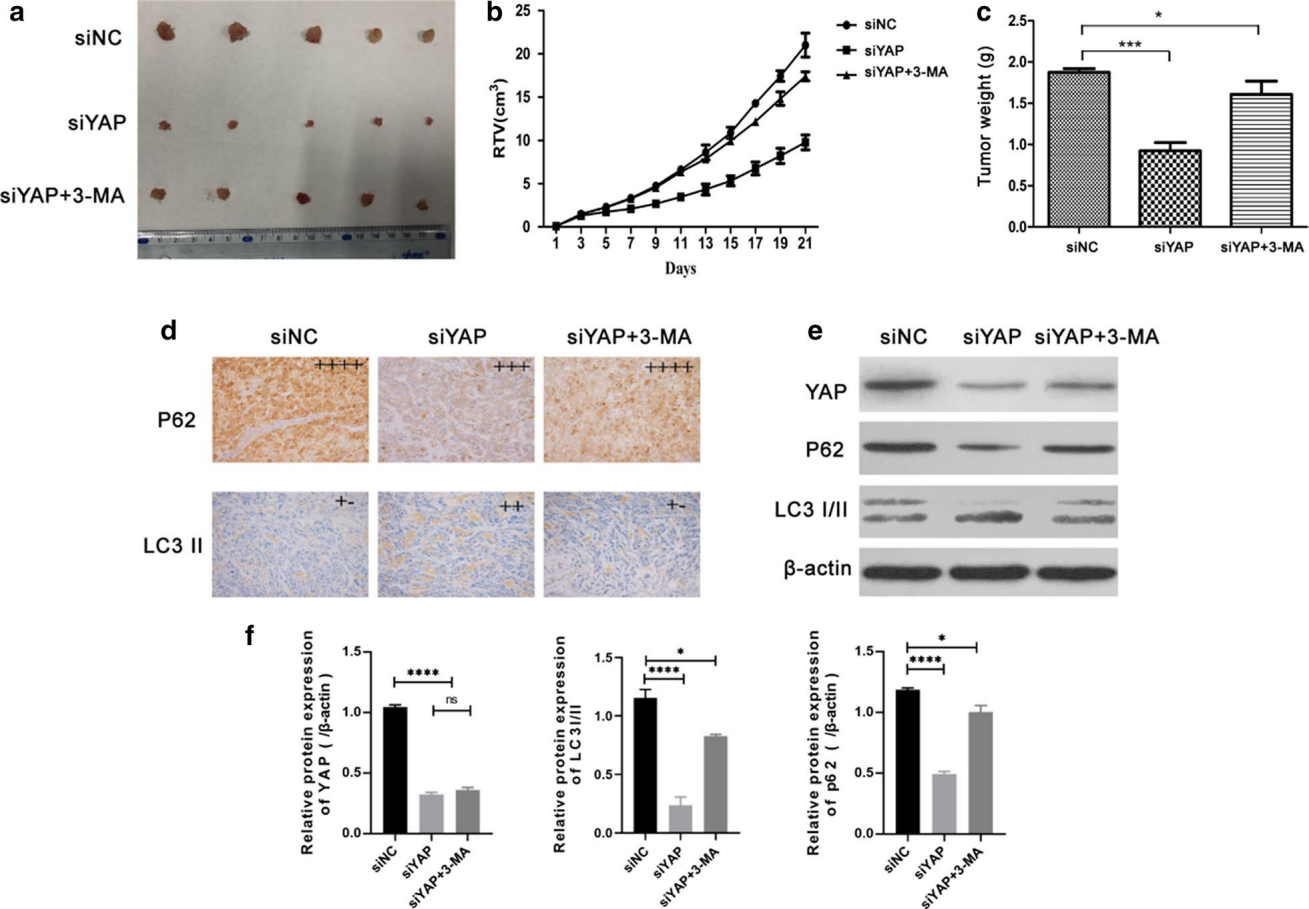
Knockdown of YAP inhibited tumor growth through autophagy in vivo. **a** Image of tumor size in A549 cells tumor xenograft treated with control, siYAP and siYAP + 3MA. **b**, **c** Represented figure indicated the tumor growth after intraperitoneal injection with or without 3MA. **d  **Immune staining indicated the expression of p62 and LC3 II, scale bar = 200 µm. **e**, **f** Western blot showed the expressive levels of YAP, p62, and LC3I/II in tumor tissues treated with or without siYAP and 3MA. Data were shown as the Mean ± SD (one-way analysis, *P < 0.05, **P < 0.01, ***P < 0.001)

We used LUAD xenograft model to observe the effects of YAP knockdown with or without 3-MA treatment at the concentration of 15 mg/Kg in vivo. A549 cells stably expressing siNC or siYAP were inoculated into nude mice, and the mice in A549-siYAP + MA group were administered with 3-MA by intraperitoneal injection every two day. On Day 21, the tumor mass of the siYap group of mice was the smallest (Fig. 6a), and the tumor volume or weight also showed the same trend (Fig. 6b, c). Immunoblotting and immunohistochemical examination of tumor tissues with staining of monoclonal anti-YAP, anti-p62, and LC3I/II antibody indicated that YAP knockdown could eventually slow down the tumor growth by promoting autophagy. Meanwhile, 3-MA could reinstate the tumorigenicity of A549-siYAP cells by impeded autophagy flux in vivo (Fig. 6d–f).

## Discussion

The Hippo pathway plays a critical role in sensing intrinsic and extrinsic signals and regulating multiple aspects of growth at both cellular and organ levels. Therefore, targeted therapies for the Hippo signaling pathway are gaining more attention. Recently, a systematic profiling of 9,125 tumor samples revealed a widespread dysregulation of Hippo pathway components in multiple human cancer types [31] The Hippo pathway integrates multiple signals to regulate the activity of core transcriptional coactivators YAP/TAZ, which directly or indirectly control multiple cancer hallmarks, including proliferation, survival, evading growth suppressors, reprogramming energy metabolism, angiogenesis, invasion and metastasis, and cancer stem cells, as well as inflammation and immunosuppression [31]. Our previous research found that TAZ was an oncogene closely associated with the therapeutic effect of cisplatin [32] and EGFR-TKIs [33, 34] in lung adenocarcinoma. However, the role of YAP in lung adenocarcinoma remains unclear. In current study, we validated YAP expression profiling in LUAD obtained from GEO and TCGA database. The results showed that YAP was significantly highly expressed in samples of LUAD patients and was associated with 5-year survival. We further clarified YAP manipulated proliferation through PTEN/AKT/mTOR-mediated autophagy in lung adenocarcinomas by enrichment analysis and mechanism research *in vitro* and in vivo.

Autophagy, known as a highly conserved physiologic process, maintains intracellular homeostasis by degrading potentially harmful cytoplasmic materials in non-selective or selective phagocytosis [35]. In the last few years, plenty of evidence has proved that autophagy, a double-edged sword in tumorigenesis, plays complex and ambiguous roles in numerous human diseases including cancer [23]. Autophagy is generally considered as a tumor-suppressing process removing hazardous metabolite and organelles to endow stress tolerance, limit general oxidative damage, and sustain viability in numerous extracellular and intracellular stresses, such as starvation and hypoxic microenvironment [36, 37]. However, the activation of autophagy could also play an important role in promoting tumor progression by enabling cancer cells to survive in harsh conditions. A recent research study has shown that the consequence of lacking autophagy in tumors induced chronic tumor cell death, which also accompanied by stimulating the release of inflammatory factor [38]. Taken together, its complex role in oncogenesis might depend on different types and genetic context of tumors.

Recently, Rubinsztein and colleagues have defined how mechanically repressed YAP/TAZ activity impacts autophagy to contribute to core phenotypes resulting from high cell confluence that are lost in various cancers [39]. Based on our previous research and biological pathway enrichment analysis of GSE64550 datasets, we speculated that the autophagy process plays a role in manipulating proliferation of YAP in LUAD. In this study, we demonstrated that the downregulation of YAP induced autophagy and then inhibited proliferation through PTEN/AKT/mTOR pathway in lung adenocarcinoma. In contrast, once autophagy flux was suppressed by 3-MA via eliminating autophagosome formation and its maturation, proliferation inhibition by siYAP was rescued. Similarly, in hepatocellular carcinoma, Youngmin et al. showed that Yap is an autophagy substrate and mediator of tissue remodeling and hepatocarcinogenesis independent of the p62/Sqstm1-Nrf2 axis [40]. Sunshen et al. suggested that YAP upregulation endowed HCC cells with multi-drug resistance via the RAC1-ROS-mTOR pathway, resulting in the repression of autophagy-related cell death [41].

In the past decade, a number of studies have revealed crosstalk between the Hippo and mTOR pathways. Karen Tumaneng and colleagues have found that YAP regulates the expression of the tumor suppressor PTEN and impinges on the PI3K/mTOR pathway that regulates cell size through the control of protein translation and autophagy. They demonstrated that tumor suppressor PTEN, an upstream negative regulator of mTOR, as a critical mediator of YAP in mTOR regulation [29]. In the present study, YAP overexpression decreased the protein level of the tumor suppressor PTEN,while YAP knockdown increased PTEN protein level. Consistently, YAP-induced PTEN loss led to AKT activation. To determine whether PTEN was a critical target of YAP in activating mTOR, PTEN was ectopically expressed in YAP-overexpressing cells by transient transfection. In parallel, PTEN was knocked down in YAP silent cells. Indeed, re-expression of PTEN antagonized the effects of YAP on pAKT and pS6K. Conversely, knockdown of PTEN in siYAP cells rescued pAKT and pS6K. We found that YAP stimulated the phosphorylation of AKT and S6K in a PTEN-dependent manner. In addition, P16 and P21 has been shown to regulate the stability of P53, and regulate the autophagy process through the P53 pathway [42–46]. Here, we confirmed that YAP affected autophagy in NSCLC cell lines in the way via independent of P53/P21/P16, and affecting tumor cell proliferation. As a result, the link from YAP - PTEN -mTOR reveals a web of signaling networks which coordinate with each other to fine-tune physiological and pathological processes.

## Conclusions

**Fig. 7:**
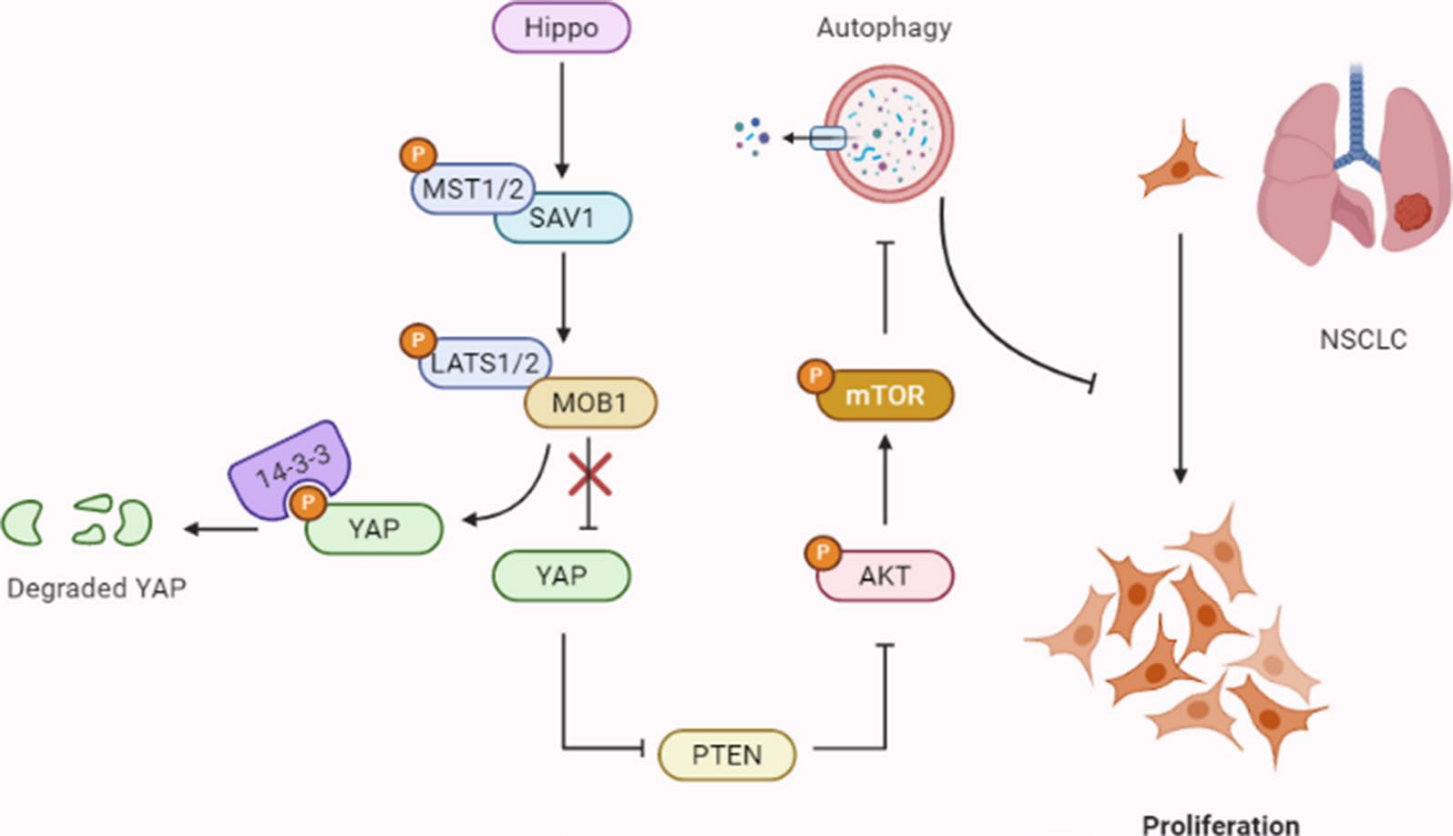
The relationship between YAP and PTEN/AKT/mTOR-mediated autophagy. When the Hippo pathway is revitalite, the activation of core Hippo pathway kinases (MST1/2, SAV1, MOB1A/B or LATS1/2) are ON, and YAP is phosphorylated and cytoplasmically retained by 14-3-3 for degradation. Above process enhancing autophagy by inhibiting AKT/mTOR pathway via elevating PTEN activation, the increase in autophagy further suppressing the non-small cell lung cancer cells proliferation. Once Hippo pathway is inactivated, YAP overexpression inhibits the activity of PTEN, which blocks the AKT/mTOR-mediated autophagy signaling pathway, promoting the proliferation of non-small cell lung cancer cells. Thus, Hippo pathway critical transcriptional coactivators YAP manipulates the proliferation of lung adenocarcinoma, which is regulated by PTEN/AKT/mTOR autophagic signaling

In conclusion, we demonstrate that Hippo pathway critical transcriptional coactivators YAP manipulates the proliferation of lung adenocarcinoma, which was regulated by PTEN/AKT/mTOR autophagic signaling. Our findings provide new insights into the underlying mechanism of Hippo-YAP pathway in lung adenocarcinoma and may help identify new potential therapeutic targeting of Hippo pathway (Fig. 7).

## Data Availability

Not applicable.

## Abbreviations

YAP: Yes-associated protein
MST1/2: Mammalian Ste20-like kinases 1/2
LATS1/2: Large tumor suppressor 1/2
SAV1: Salvador Family WW Domain Containing Protein 1
MOB1A/B: Mps One Binder Kinase Activator-Like 1A/B
TEAD: TEA domain family member
EMT: epithelial-to-mesenchymal transition
LUADs: Lung adenocarcinomas
3-MA: 3-Methyladenine
